# Molecular Simulation Elaborating the Mechanism of 1β-Hydroxy Alantolactone Inhibiting Ubiquitin-Conjugating Enzyme UbcH5s

**DOI:** 10.1038/s41598-019-57104-4

**Published:** 2020-01-10

**Authors:** Youdong Xu, Xianli Meng

**Affiliations:** College of Pharmacy, Chengdu University of Traditional Chinese Medicine, Chengdu, 611137 P.R. China

**Keywords:** Biochemistry, Computational biology and bioinformatics, Molecular biology, Structural biology

## Abstract

1β-hydroxy alantolactone, a sesquiterpene lactone, exhibits potent anti-inflammatory and anticancer activities. Recently, it has been found to target UbcH5s by covalently bonding with Cys85 specifically, but the exact molecular basis remains unclear. Here, we analyzed the structural specificity of the catalytic site of UbcH5s by comparing them with other E2 proteins. Molecular dynamics was performed to detect the structural stability of the catalytic site. Docking method was then used to predict conformations of ligand docked at the catalytic site of UbcH5s. The electrostatic surface and charge distribution of ligand and proteins were analyzed by quantitative calculation. Molecular dynamics was used to detect the stability of docking complexes of 1β-hydroxy alantolactone and UbcH5s, the covalently bonded intermediates and the products. The QM/MM methodology was used to calculate the free energy barrier of hydrogen transfer and formation of covalent bond between 15-position carbon of ligand and Cys85. Results revealed that the structure of the catalytic site is stable, and 1β-hydroxy alantolactone can dock at the catalytic site with correct conformation. Molecular dynamics further demonstrates that 1β-hydroxy alantolactone can steadily combine with UbcH5s. Intermediate and product of catalytic reaction are also certified to be stable. Besides, Asp112 and Asn114 function as anchors to fix ligand, ensuring it steadily docked at catalytic site to complete covalent reaction. More importantly, we have found that Cys85 of UbcH5c is more efficient to form a covalent bond with the ligand in comparison with UbcH5a and UbcH5b. Our results successfully explained the mechanism of 1β-hydroxy alantolactone covalently bonding with UbcH5s. Such molecular mechanism may provide a better insight into the molecular development or modification for ubiquitin-related drugs.

## Introduction

Recent studies have indicated that medicinal herbs have been gradually widely used despite insufficient information correlating with their mechanism of action. At present, research institutions or companies are devoting themselves to discovery a class of bioactive compounds, their valuable target proteins and mechanisms of action against diseases. Sesquiterpene lactones are plant-derived bioactive constituents, which have subjected numerous studies. Researches revealed that they could be used in medicines against inflammation, cancer, malaria, and viral and bacterial infection^1,2^. Pharmacodynamic studies further reported that sesquiterpene lactones might exert therapeutic effects by inhibiting NF-κB and MAPK pathway activation^3–8^. Nevertheless, the molecular mechanism of sesquiterpene lactones targeting protein on NF-κB and MAPK pathway is unknown.

**Figure 1:**
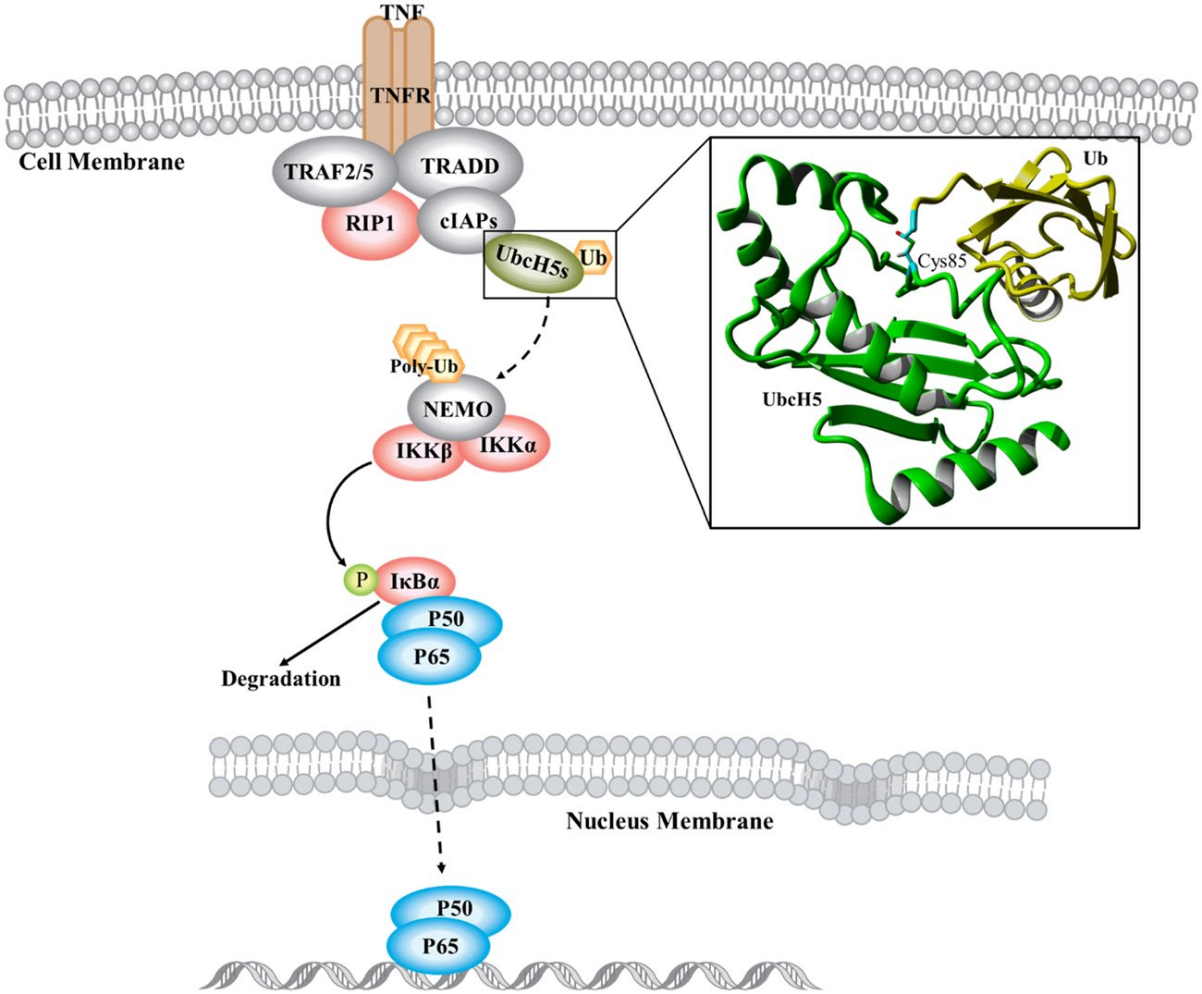
The cell signal pathway of TNF-α/NF-κB. TNF-α could dock with TNF receptor, and recruit TNF receptor-associated death domain protein (TRADD), TNF receptor-associated factor 2 (TRAF2), the cellular inhibitor of apoptosis proteins 1 and 2 (c-IAP1 and c-IAP2), and receptor-interacting protein 1 (RIP1). Then UbcH5s carrying Ub will mark NF-κB essential modulator (NEMO) with multi-step. IKKs phosphorylates the NF-κB inhibitor IκB and induce proteasomal degradation. Finally, NF-κB will transfer into the nucleus to participate in transcription. Pink ellipse is kinase, Ub is yellow hexagon, UbcH5s are dark green ellipse, phosphatase is light green circle, NF-κB is bright blue ellipse. Dotted arrow is multistep stimulatory modification. UbcH5 conjugated with Ub are also showed as 3D structure, green strip structure is UbcH5, yellow strip structure is Ub, Cys85 and covalent bond are showed as stick model.

1β-hydroxy alantolactone that belonged to sesquiterpene lactone has been found to possess anti-inflammatory and anticancer activities. From the data of its discovery, studies reported almost the same pharmacological effects of 1β-hydroxy alantolactone and its derivatives^9^, of which they can covalently bond with Cys38 of p65, further influencing NF-κB signal pathway^10–12^. However, these results failed to explain whether 1β-hydroxy alantolactone has specific selectivity for Cys38, nor can it explain the mechanism of action between the ligand and the protein. Recently, Zhenlin Hu and Weidong Zhang team’s study^13^ found that 1β-hydroxy alantolactone can specifically establish covalent bonds with Cys85 of Ubiquitin-conjugating Enzyme H5 (UbcH5), preventing catalytic reaction and inhibiting effectively activation of TNF-α/NF-κB pathway (the pathway is shown in Fig. 1). Although preliminary docking simulation in their research has been performed, the exact molecular basis remains unclear. To modify the structure of 1β-hydroxy alantolactone and better develop this leading compound against UbcH5 in the treatment of inflammatory diseases, the specific mechanism of 1β-hydroxy alantolactone interacting with UbcH5s is needed to explore in-depth.

Ubiquitination is a hierarchical enzymatic cascade, during which a ubiquitin (Ub)-activating enzyme (E1) first activates Ub and transfers it to Ub-conjugating enzyme (E2), then E2 interacting with Ub ligase (E3) transfers Ub to the lysine residue of target proteins^14^. UbcH5s belonged to E2 enzymes are involved in TNF-α/NF-κB signaling pathway, consisting of three homologs‒UbcH5a, UbcH5b, and UbcH5c^15^. UbcH5s are proven to cooperate with c-IAPs to generate a linear polyUb chain on the NF-κB essential modifier (NEMO)^16–18^. Moreover, UbcH5s knockdown can significantly influence the ubiquitination of NEMO, IKK activation, and NF-κB-regulated gene transcription. Therefore, UbcH5s are essential protein modulating TNF-α/NF-κB activity, which might be a valid target in treating inflammation.

Molecular dynamics (MD) simulation is used to predict how every atom in a protein or other molecular system will move over time based on Newton’s physics^19^. These simulations can capture a wide variety of important biomolecular processes, including conformational change, ligand binding, and protein folding. Importantly, such simulations can also predict how biomolecules will respond—at an atomic level—to perturbations such as mutation, phosphorylation, protonation, or the addition or removal of a ligand. MD simulations are often used in combination with experimental structural biology techniques, including X-ray crystallography, cryoelectron microscopy (cryo-EM) and nuclear magnetic resonance (NMR). During MD simulations, a variety of properties, such as the flexibility of ligand or residues, hydrogen bonds, and hydrophobic property, can be analyzed through successive trajectories of molecular dynamics. According to these properties, explaining the mechanisms of ligand-protein interactions becomes possible. Up to now, this approach is widely applied in biomolecules and drug discovery^20–22^. However, the limitation for this force-field based simulations is the chemical reactivity, such as bond break or formation, which cannot be simulated during MD. To solve this problem, hybrid quantum mechanics/molecular mechanics (QM/MM) methods have been developed^23^. QM/MM methods combine a quantum mechanical treatment of the subset of atoms involved in the chemical reaction with a molecular mechanics description of the surroundings. Partitioning the system is based on the idea that the apparent redistribution of the electron is often limited to a small subset of the atoms, while the effects of the majority atoms in the system can be described adequately by a classical treatment of intermolecular interactions. Therefore, QM calculation with higher accuracy is applied to treat the formation of a covalent bond at the catalytic site, and a classical (MM) treatment is used for the surroundings in this research.

In this research, we utilized MD simulations and QM/MM calculations to describe comprehensively the reaction process of 1β-hydroxy alantolactone catalyzed by UbcH5s. Here, Cys85 is a conservative catalytic site and activated when thiol hydrogen transfers to Asp117. The activated Cys85 will undergo a nucleophilic attack on the carbon atom of α-methylene of the ligand and form a covalent bond with it. Asn114 and Asp112 are found to form stable hydrogen bonds with substrate to guarantee it combining firmly with protein at the active site. Besides, Cys85 of UbcH5c is more efficient to be activated and form a covalent bond with the ligand in comparison with UbcH5a and UbcH5b. 1β-hydroxy alantolactone might be more selective for UbcH5c. In this research, we explain the reaction mechanism of 1β-hydroxy alantolactone interacting with UbcH5s for the first time, hoping the information that could assist the modification of molecule and drug development against inflammation.

## Results

### Sequence alignment and structure superposition analysis

**Figure 2:**
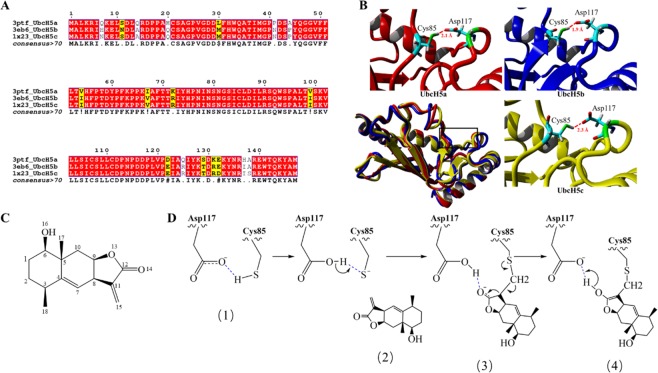
(**A**) Sequence alignment of UbcH5s; (**B**) 3D structural comparison of UbcH5s, superimposed structures are displayed in the lower left corner, separate structure is shown around it. Proteins are showed as strip model, Cys85 and Asp117 showed as stick model, hydrogen bonds are showed as red dash lines; (**C**) The 2D structural formula is numbered by ChemDraw ultra 2.0. (**D**) The mechanism scheme of Cys85-Asp117 catalyzing 1β-hydroxy alantolactone.

Human UbcH5s (UbcH5a^24^, UbcH5b^25^ and UbcH5c^26^) sequence alignment and structural superposition were performed using CLUSTALW^27^, ESPript 3.0^28^ and PyMol 1.6^29^ (as shown in Fig. 2A,B). According to alignment analysis, the homology of the three proteins is up to 99.8%, and the amino acid sequences of the three proteins are almost identical. The backbone atoms were used to compute structural superposition. The ΔRMSD of three superposed tertiary structures is 0.98 Å, indicating that the three-dimensional structure of the proteins is almost the same. Cys85 responsible for covalently binding with the substrate is conservative in all three proteins. Asp117 near 1.6–1.8 Å of Cys85 can be found, which can form Cys-Asp catalytic diad. Meanwhile, Cys85 can form a hydrogen bond with Asp117, and the hydrogen-bond distances are respective 2.1, 1.9 and 2.3 Å. However, the similar paired residues and the hydrogen bonds are not found in other E2 protein (as shown in Figure S1), suggesting that Cys85-Asp117 might be a unique catalytic diad in UbcH5s.

The structural formula of 1β-hydroxy alantolactone is shown in Fig. 2C, and we have put forward a catalytic mechanism of 1β-hydroxy alantolactone forming a covalent bond with UbcH5s, as shown in Fig. 2D. At the beginning of the catalysis, the strong hydrogen bond between Cys85 and Asp117 is formed, and the hydrogen-bond distance should be maintained stable **(1)**. Then hydrogen of Cys85 thiol will transfer to carboxyl oxygen of Asp117, resulting in activation of Cys85. The activation of Cys85 will make a nucleophilic attack on the 15-position carbon of 1β-hydroxy alantolactone docked at the catalytic site **(2)**. Subsequently, Cys85 will form a covalent bond with the ligand, and the electron will migrate to the 14-position oxygen of ligand because of the attraction of oxygen atoms to electrons. Simultaneously the 14-position carbonyl oxygen of ligand will form a hydrogen bond with the carboxy hydrogen of Asp117 **(3)**. Finally, the hydrogen of Asp117 carboxyl should be transferred to the 14-position carbonyl oxygen of ligand, and the chemical double bond is rearranged. Asp117 should still form a hydrogen bond with the substrate when Michael’s addition reaction is completed **(4)**.

### Structural stability of catalytic site of UbcH5s

**Figure 3:**
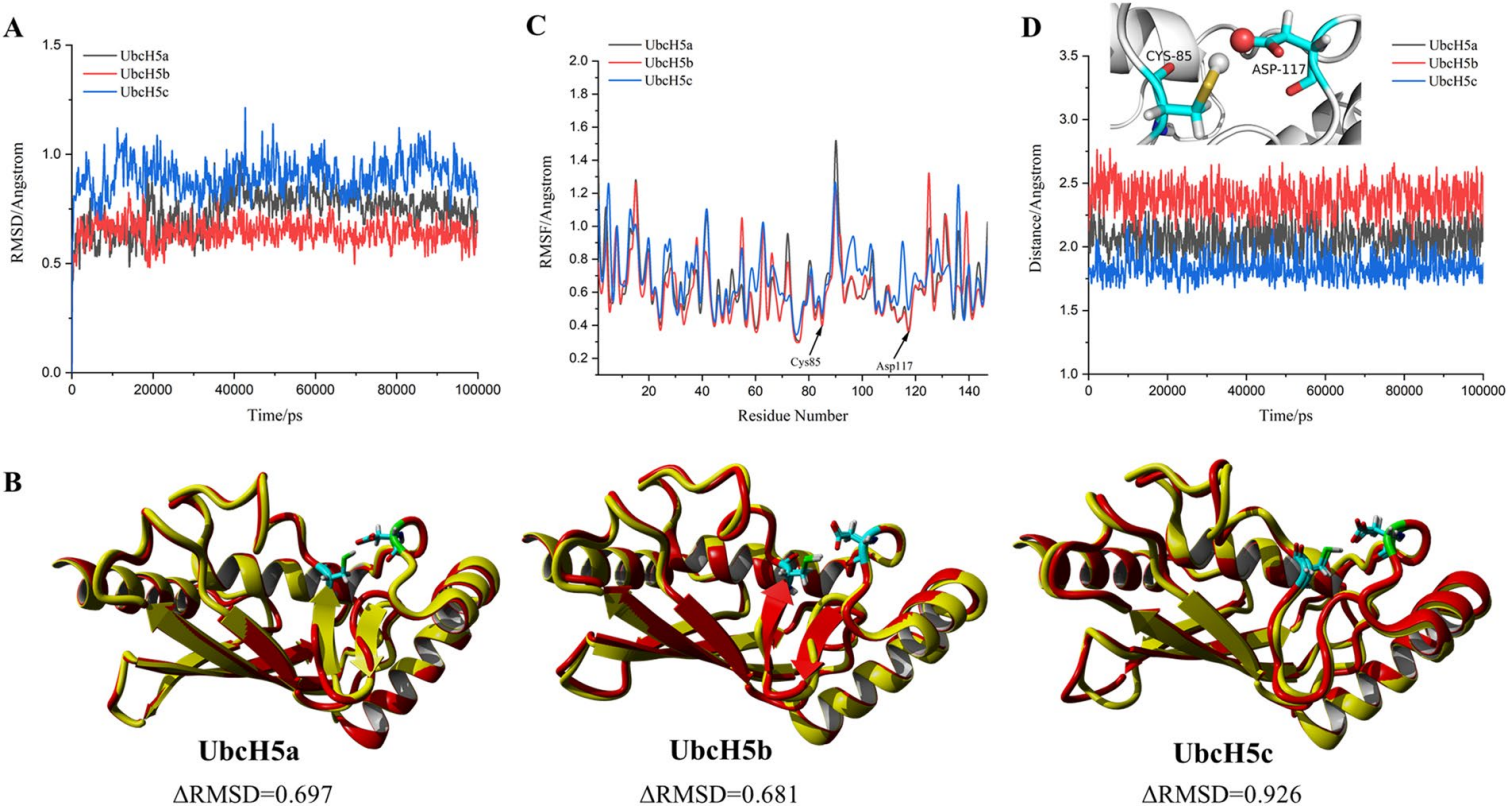
(**A**) Backbone RMSD of the entire protein of UbcH5a/b/c; (**B**) Superposed conformations between initial and 100 ns structures of UbcH5a/b/c, the yellow strip models are initial structures and red strip models are structures at 100 ns; (**C**) RMSF of all residues in UbcH5a/b/c; (**D**) The change of distance between carboxyl oxygen of Asp117 and thiol hydrogen of Cys85 in UbcH5a/b/c, the two residues are showed as stick model and two atoms are showed as spherical model.

At the **(1)** step of the catalytic process, the 100 ns duration simulations were performed to detect whether the protein structures are stable during the simulation. The global structural stability of UbcH5s was evaluated using the RMSD of the backbone atoms (Fig. 3A). During the 100 ns simulation, RMSDs of UbcH5s fluctuate between 0.5 and 1.5 Å. The ΔRMSDs of UbcH5s are all less than 2 Å, which indicate the stability of structures throughout the simulation. In Fig. 3B, a superimposed analysis of backbone atoms between the initial and final configurations of UbcH5s for molecular dynamics was performed, and ΔRMSD < 1 Å. It is worth noting that the 3D positions of Cys85 and Asp117 in initial and final snapshots are almost entirely coincident, and there is not displacement observed. According to this analysis, the catalytic sites of UbcH5s are stable when the substrate not bound.

After the determination of global structural stability by RMSD, we performed a more detailed analysis of each amino acid in UbcH5s. As shown in Fig. 3C, RMSF was used to evaluate the flexibility of the residues. The RMSF values Cys85 and Asp117 are found to be at the bottom of the curve, which suggested that these two essential amino acids with small fluctuation are beneficial to the progress of the catalytic process.

In Fig. 3D, the distance between the carboxyl oxygen of Asp117 and thiol hydrogen of Cys85 is maintained at around 2.0 Å in UbcH5a, 2.4 Å in UbcH5b and 1.75 Å in UbcH5c. In comparison with UbcH5a and UbcH5b, the distance between Cys85 and Asp117 in UbcH5c is closer, suggesting that a stronger hydrogen bond might be formed.

### Interactions between 1β-hydroxy alantolactone and UbcH5s

**Figure 4:**
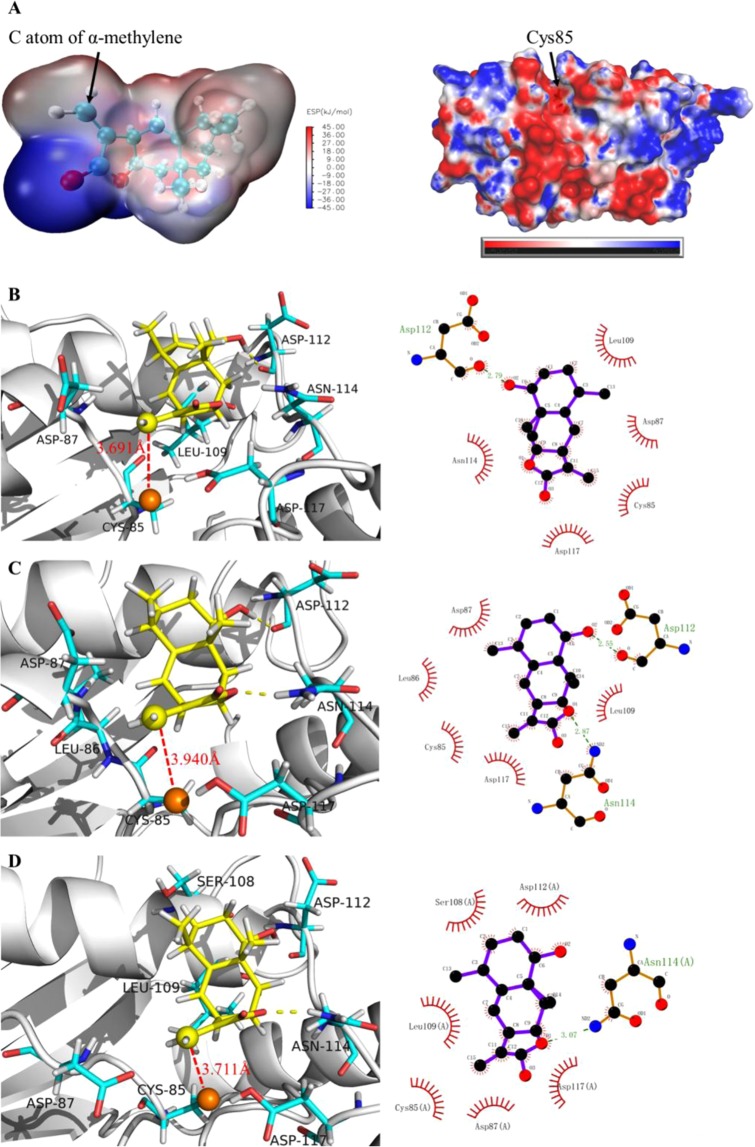
(**A**) Surface electrostatic potential analysis for ligand and protein. The reddish area represents positive charge and biased blue area negative charge for ligand. But for protein, the reddish area represents negative charge and biased blue area positive charge; (**B**–**D**) The left side is the 3D schematic diagram of the docking conformations and the interacting residue of UbcH5a/b/c, and the right side is the corresponding 2D-interaction analysis. Ligand is showed as yellow stick model, protein showed as white strip model, interacting residues showed as cyan stick model, and 15-position carbon of ligand as well as sulfur atom of Cys85 showed as spherical model. Hydrogen bond is showed as dotted line. In 2D interaction diagram, ligand is showed as purple ball-and-stick model, residues forming hydrogen bond is also showed as ball-and-stick model. Hydrogen bond is showed as green imaginary line and hydrophobic residues appear as arc-like lashes.

**Figure 5:**
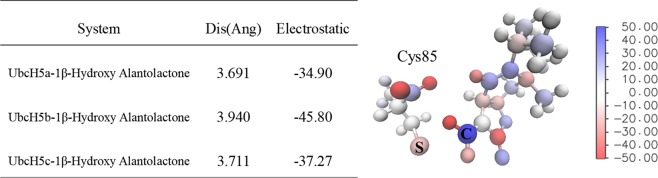
Result of energy decomposition, the electrostatic interacting energy between 15-position carbon of ligand and sulfur of Cys85 is listed. On the is the ball and stick model of ligand and Cys85, this model is colored according to charge, and the blueish atom has a positive charge and the reddish atom has a negative charge.

At the **(2)** step of the catalytic process, molecular docking techniques were used to predict the docking mode of 1β-hydroxy alantolactone to macromolecular partner, UbcH5s. The best matching conformations of ligands docked with activated catalytic sites of UbcH5s are shown in Fig. 4. Because of the covalent attachment of ligand and protein is Michael addition, electrostatic potential surfaces (ESP) of ligand and protein were analyzed firstly (as shown in Fig. 4A). The ESP of 15-position olefin carbon of ligand is 9 kJ/mol, in which atom is positively charged. The sulfur atom of Cys85 in the catalytic site is shown as negatively charged. This indicated that these two atoms can form electrostatic interactions, and subsequent docking poses could further reinforce this hypothesis. Figure 4B–D display the best docking pose of ligand, from which 1β-hydroxy alantolactone enables to dock at the catalytic site. The 15-position olefin carbon of ligand facing the sulfur atom of Cys85 and the distances between two atoms are respectively 3.691, 3.711 and 3.940 Å in UbcH5s. In addition, combining the 2D scheme of docking results, 1β-hydroxy alantolactone is detected to form hydrogen bonds with Asp112 and Asn114. The two crucial hydrogen bonds can enhance the ligand binding at the catalytic site. In Fig. 5, energy decomposition between ligand and around residues was performed to confirm again that 15-position carbon of ligand can still form strong electrostatic interaction with Cys85, and the interaction is not affected by surrounding residues.

### Stability of docking complex system during molecular dynamics simulation

**Figure 6:**
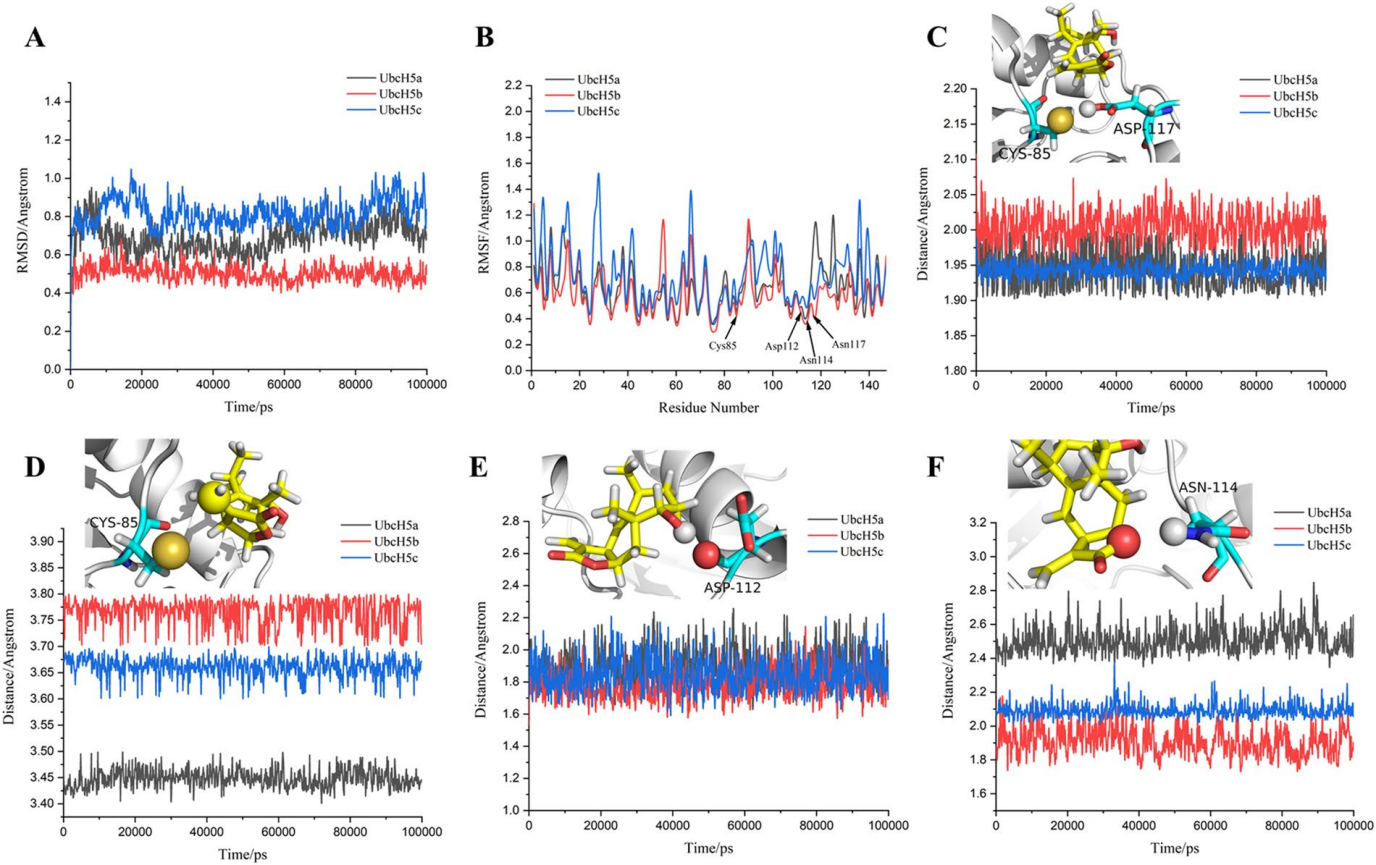
(**A**) Backbone RMSD of UbcH5a/b/c docked with ligand; (**B**) RMSF of every residue in UbcH5a/b/c; (**C**–**F**) The change of distance between carboxyl hydrogen of Asp117 and sulfur atom of Cys85, carbon at 15 position of ligand and sulfur atom, hydroxyl hydrogen at 16 position of ligand and oxygen of Asp112, and epoxy at 13 position and amino hydrogen of Asn114. These detected atoms are showed as spherical model, rest residues are showed as stick model and proteins showed as strip model.

During the **(2)** step of the catalytic process, 100 ns MD simulations for ligand-UbcH5s complex were performed. RMSD values of the backbone atoms were calculated, as shown in Fig. 6A. The RMSD values of all complexes fluctuate between 0.4–1.0 Å, and ΔRMSDs of all complexes obviously is less than 2 Å. The result indicates that all complexes keep equilibrium during 100 ns simulation.

The analysis of RMSF (as shown in Fig. 6B) reveals that the values of Cys85, Asp112, Asn114 and Asp117 are at the bottom of the curve. According to the above docking results, Cys85, Asp112 and Asn114 can respectively form electrostatic interaction and hydrogen bonds with the ligand. Asp117 can form a hydrogen bond with Cys85. These residues that have minimal fluctuations are conducive to the immobilization of 1β-hydroxy alantolactone docked at the catalytic site.

In Fig. 6C–F, the distances between sulfur atom of Cys85 and hydroxyl hydrogen of Asp117 are probably maintained at 1.95, 2.00 and 1.95 Å; the 15-position carbon of ligand and sulfur atom of Cys85 are about 3.45, 3.77 and 3.67 Å; the distances between 16-position hydroxyl hydrogen of ligand and oxygen of Asp112 are around 1.6–2.1 Å; the distances between 13-position epoxy of ligand and amino hydrogen of Asn114 are about 2.45, 1.85 and 2.1 Å. These stable distance changes further prove that ligand can stably bind at the catalytic site.

As described above, Asp112 and Asn114 can respectively form hydrogen bonds with 16-position hydroxyl hydrogen and 13-position epoxy of ligand (as shown in Figure S2A), from which the number of hydrogen bonds is almost 2. During 100 ns simulation, the hydrophobic strength between ligand and UbcH5s is detected, and the values fluctuate from 6 to 9 (as shown in Figure S2B). More importantly, MM-PBSA per frame was calculated, and the bind-energy between ligand and UbcH5s is about −50 kJ/mol, −40 kJ/mol and −60 kJ/mol (as shown in Figure S2C). According to these results, ligand could be dock steadily at catalytic sites of UbcH5s.

### Covalent bond formation and stability of complex system

**Figure 7:**
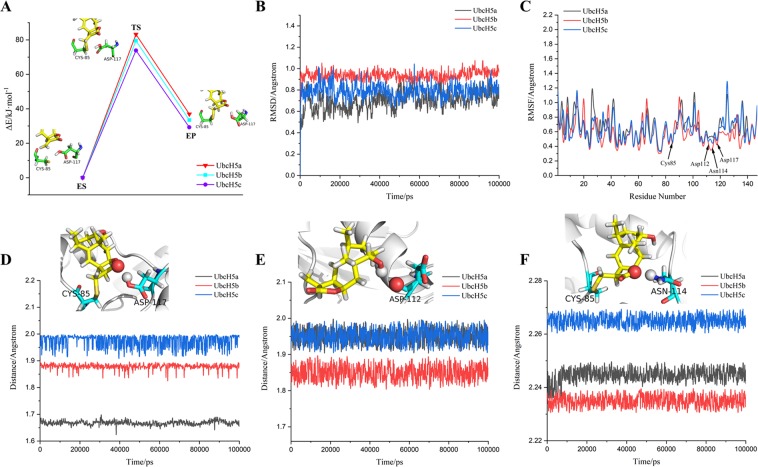
(**A**) Free energy barriers for 1β-hydroxy alantolactone forming covalent bond with Cys85 of UbcH5s using QM(B3LYP)/MM-UFF. (**B**) Backbone RMSD of UbcH5a/b/c covalently bonded with ligand; (**C**) RMSF of all residues in UbcH5a/b/c; (**D**–**F**) The change of distance between carboxyl hydrogen of Asp117 and 14-position oxygen of ligand, 16-position hydroxyl hydrogen and oxygen of Asp112, and 13-position epoxy and amino hydrogen of Asn114. These detected atoms are showed as spherical model, rest residues are showed as stick model and proteins showed as strip model.

At the **(3)** step of the catalytic process, we calculated the free energy barriers for 1β-hydroxy alantolactone covalently bonding with Cys85, and the 10 energy barriers for each UbcH5a/b/c system are showed in Figure S3A. From the initial substrate complex (ES) to transition state (TS), the energy barriers of UbcH5a are in the range of 80–100 kJ/mol, UbcH5b 70–85 kJ/mol and UbcH5c 67–77 kJ/mol. According to the calculated results, Savitzky–Golay^30^ method was used to generate the energy barrier curve, which can increase the precision of the data without distorting the signal tendency. As shown in Fig. 7A, the free energy barriers between ES and TS are respectively 83.22, 79.55 and 73.86 kJ/mol.

Due to the attraction of 14-position oxygen of ligand to electrons, the electric displacement has occurred. In order to determine our speculation, we have detected the distribution of electrons by calculating the Mulliken charge. As shown in Figure S4, the charge of 11-position carbon is respectively −0.165591, −0.170231 and −0.178964 in UbcH5a/b/c. The charge of 14-position oxygen is respectively −0.469924, −0.473256 and -0.496315 in UbcH5a/b/c. Combining with the analysis of free energy barriers, UbcH5c might be easier to accept hydrogen and complete electron transfer to Asp117.

Subsequently, molecular dynamics was used to detect the reliability and stability of the intermediates. The RMSD of all complexes fluctuates between 0.6–1.0 Å, and ΔRMSDs of all complexes obviously is less than 2 Å. The result shows that complexes keep equilibrium during 100 ns simulation (as shown in Fig. 7B). The RMSF analysis was performed to evaluate the flexibility of all residues (as shown in Fig. 7C). The fluctuations of Cys85, Asp112, Asn114 and Asp117 are still at the bottom of the curve, which reflects the stability of these residues. In Fig. 7D–F, the distances between the 14-position carbonyl oxygen of ligand and hydroxyl hydrogen of Asp117 are maintained at around 1.67, 1.88 and 1.99 Å, 16-position hydroxyl hydrogen and oxygen of Asp112 at 1.95, 1.85 and 1.95, and the 13-position epoxy and amino hydrogen of Asn114 at 2.24, 2.23 and 2.27 Å. The ligand is found to form hydrogen bonds with Asp112, Asn114 and Asp117. The hydrogen bonds content between these atoms almost maintains at around 3 (as shown in Figure S2D). The hydrophobic strength between ligand and UbcH5s are detected to change from 7 to 9 (as shown in Figure S2E). MM-PBSA per frame was calculated, and the bind-energy between ligand and UbcH5s is about −557 kJ/mol, −556 kJ/mol and −568 kJ/mol (as shown in Figure S2F). Since **(3)** step is an essential procedure that generates an intermediate, and the intermediate determines whether the catalytic speculation process is reasonable. According to the above results, the stability of the intermediate further verify our inferred reaction mechanism and confirms that our characterized transition state is meaningful.

### Hydrogen atom transfer and stability of complex system

**Figure 8:**
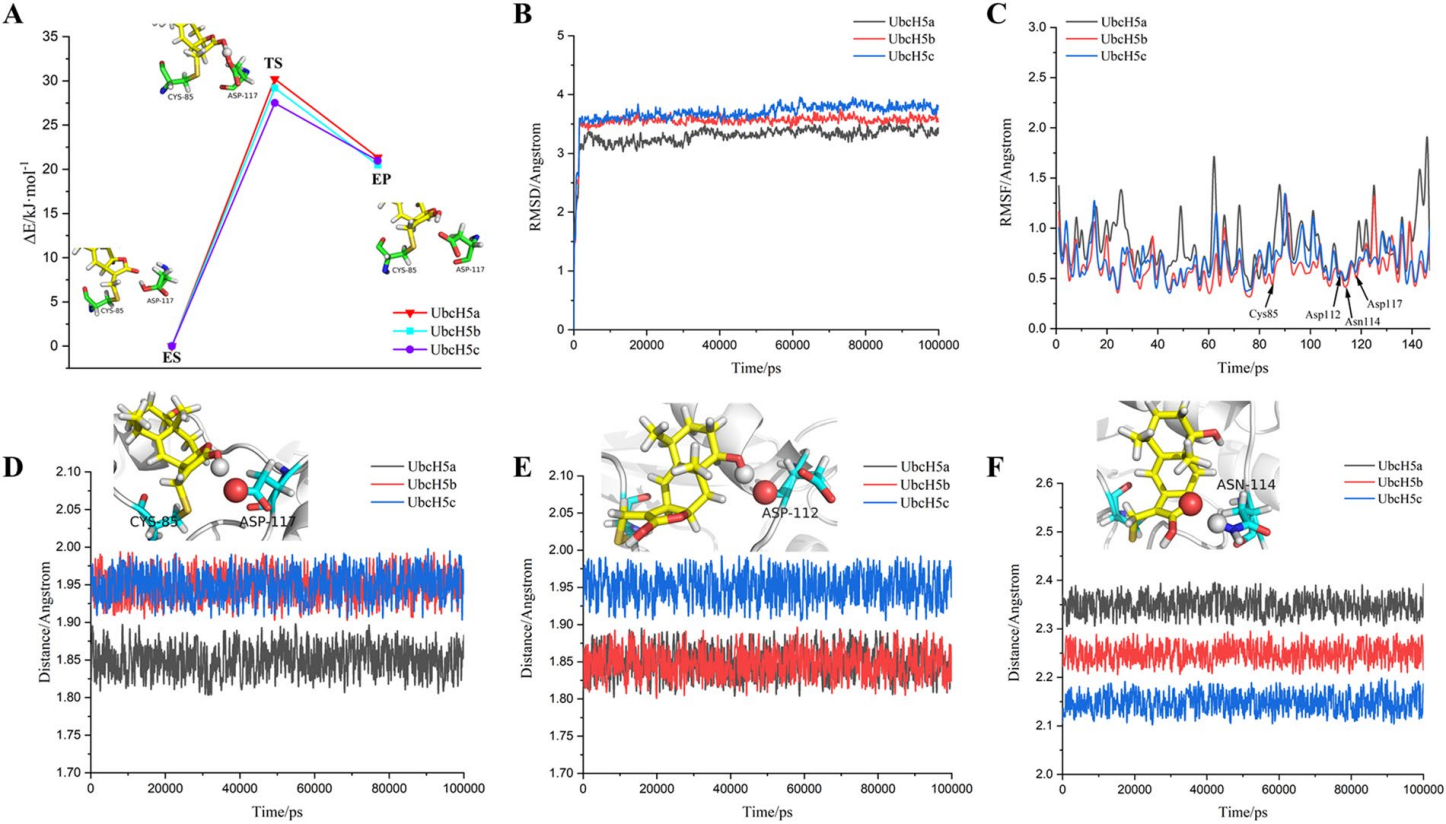
(**A**) Free energy barriers for hydrogen transferring from Asp117 to oxygen at 14 position of ligand using QM(B3LYP)/MM-UFF. (**B**) Backbone RMSD of UbcH5a/b/c covalently bonded with restored ligand; (**C**) RMSF of all residues in UbcH5a/b/c; (**D**–**F**) The change of distance between carboxyl oxygen of Asp117 and 14-position hydroxyl hydrogen of ligand, 16-position hydroxyl hydrogen and oxygen of Asp112, and 13-position epoxy and amino hydrogen of Asn114. These detected atoms are showed as spherical model, rest residues are showed as stick model and proteins showed as strip model.

The **(4)** step of the catalytic process is the final procedure in which hydrogen should transfer from Asp117 to the 14-position carbonyl oxygen of ligand. The 10 free energy barriers of hydrogen transfer for each UbcH5a/b/c system are showed in Figure S3B. From the ES to TS, the energy barriers of UbcH5a are in the range of 28–31 kJ/mol, UbcH5b 28–30 kJ/mol and UbcH5c 25–29 kJ/mol. According to the calculated results, Savitzky–Golay method was used to generate energy barrier curves. As shown in Fig. 8A, the free energy barriers between ES and TS are respectively 30.21, 29.20 and 27.50 kJ/mol.

Then molecular dynamics was used to detect the reliability and stability of the products. The RMSDs of all complexes significantly raise, but ΔRMSDs of all complexes obviously is less than 2 Å, and all complexes keep equilibrium during 5–100 ns simulation (as shown in Fig. 8B). The RMSF values of Cys85, Asp112, Asn114 and Asp117 are still located at the bottom of the curve (as shown in Fig. 8C).

Figure 8D–F show the changes in distances between ligand and the above residues. The distances between the 14-position hydroxyl hydrogen of ligand and carbonyl oxygen of Asp117 are maintained probably at 1.95, 1.95 and 1.86 Å; the 16-position hydroxyl hydrogen of ligand and oxygen of Asp112 at 1.85, 1.85 and 1.95; and the 13-position epoxy of ligand and amino hydrogen of Asn114 at 2.35, 2.25 and 2.15 Å. The number of hydrogen bonds between the ligand and these atoms was analyzed (as shown in Figure S2G), from which the number of hydrogen bonds almost maintains at around 3. The hydrophobic strength between ligand and UbcH5s are detected to fluctuate from 6 to 9 (as shown in Figure S2H). The bind-energy between ligand and UbcH5s is about −535 kJ/mol, −538 kJ/mol and −545 kJ/mol (as shown in Figure S2I). These results prove the stability of the products and support our speculation for the mechanism at the **(4)** step.

### Alanine scanning mutagenesis

Hot spots residues at the protein-ligand interfaces in all complexes of 1β-hydroxy alantolactone with UbcH5s were identified through ABS-Scan. The energetic contribution of each residue involved in the interaction with the protein and ligand was evaluated by AutoDock score (kJ/mol). We extracted dynamic trajectories for alanine-scanning simulation every 5 ns (as shown in Figure S5). Upon analysis, we found Cys85, Asp112 and Asn114 with lower energy compared with other residues involved in this mutation analysis. The curves of energy contribution demonstrate the importance of these three residues in catalyzing and immobilizing ligands again.

## Conclusions

Ubiquitination plays an essential role in almost cellular process, such as cell-cycle progression, endocytosis and trafficking, and even immune-signal transduction^31^. TNF-α/NF-κB pathway has been reported to correlate with inflammatory diseases. UbcH5s are supported to play a pivotal role in TNF-α-mediated NF-κB activation by growing evidence^18,32,33^. 1β-hydroxy alantolactone since discovered is rarely reported about its pharmacological action, and only a few of literature indicated that it has anti-inflammatory effects. Apart from this, there is not a description in detail about its pharmacological mechanism. Although 1β-hydroxy alantolactone is discovered to form a covalent bond with UbcH5s^13^ effectively blocking the TNF-α/NF-κB pathway, molecular mechanism about it combined with UbcH5s was just preliminarily explored by docking method. In this research, we first elaborate how 1β-hydroxy alantolactone is covalently bonded to UbcH5s through hybrid methods including docking, MD simulations, and QM/MM calculations, which can provide the interaction mechanisms at the molecular level^34^.

Previous researches reported that Cys85 is the E2 active site residue, and it is able to form thiol bond with Ub from E1^25,35^. Meanwhile, this residue participates in transducing the structural changes at the E3-binding site to the active site of UbcH5^36^. Consistent with these findings, 1β-hydroxy alantolactone has been proved to covalently bond with cys85 of UbcH5s effectively suppressed protein activation. Therefore, we focused our attention on this residue in order to find out the mechanism of 1β-hydroxy alantolactone, specifically interacting with UbcH5s. In UbcH5s family, the sequences of UbcH5a/b/c are highly similar, and the 3D structure of these proteins almost coincide with each other. Therefore, we can assume that these proteins could perform similarity in behavior of catalysis. However, compared with other proteins such as Ubc1, Ubc7, UbcH7, UbcH8, Ube2b, Ube2j2, Ube2s, Ube2w, CDC34 and so forth, not only are there differences in sequences, but the protein structures are also vastly different. During analyzing the residues around Cys85 of UbcH5s, we found that Asp117 can form catalytic diad with Cys85. However, no Asp corresponding to Asp117 of UbcH5s is not detected in these proteins, in other word there is not Asp assisting Cys to complete catalytic reaction. This difference might be one of the reasons that 1β-hydroxy alantolactone can specifically target UbcH5s.

Because there are no obvious pocket areas on the protein surface for ligands docking, the binding poses to proteins are particularly important. Direct covalent docking experiment without judgment will make an unreasonable conformation. The research^13^ confirmed that 1β-hydroxy alantolactone can covalently modify the active site cysteine (Cys85) of UbcH5s. Therefore the ligand that could dock toward for Cys85 with correct conformation or not will determine the success or failure of the simulation experiment. We successfully obtained the pose of 15-position carbon of 1β-hydroxy alantolactone docked to Cys85 with proper orientation and distance. Moreover, the subsequent electrostatic surface analysis and energy decomposition further demonstrate the electrostatic interaction between sulfur atoms and carbon atoms. Such noncovalent interactions might serve as an initial site-recognition step when the ligand binds with UbcH5s. Then the probability of the covalent reaction and stability of the covalent adduct between ligand and Cys85 of UbcH5 should be raised.

Although molecular docking method successfully generated the suitable pose of 1β-hydroxy alantolactone interacting with UbcH5s, the conformation obtained by molecular docking is only a static result. Whether the structure can stably bind to the catalytic site over time requires further confirmation by molecular dynamics. In order to guarantee the scientific rationality of the simulation, we pay more attention to the stability of structures, residues and distances. In UbcH5s, a stable hydrogen-bond distance between the thiol of Cys85 and the carboxyl oxygen of Asp117 is beneficial to the deprotonation of Cys85 and activated Cys85 can attack the substrate nucleophilically. Therefore, we need to first evaluate the stability of the hydrogen bond distance between these two residues at the beginning of the entire simulation. Subsequently, activated Cys85 forms a covalent bond with the 15-position olefin carbon of the ligand, and the negatively charged ligand as an intermediate should form a hydrogen bond with Asp117 by 14-position oxygen atom. This hydrogen bond not only strengthens the intermediate but also to equilibrate the charge. Only stable intermediates can ensure the smooth progress of the catalysis, and it is also able to prove the correctness of our speculative reaction mechanism. When the catalytic process is completed, the 14-position carbonyl oxygen should be converted to a hydroxyl group and remains hydrogen bonding with Asp117, which can facilitate the stability of the covalently bound ligand at the catalytic site. In addition, Asp112 and Asn114 are found to form hydrogen bonds with 13-position epoxy and 16-position hydroxyl. These two hydrogen bonds are stable throughout the molecular dynamics. Since there are no obvious pockets or recessed areas on the surface of UbcH5s, hydrogen bonding plays a particularly important role in the process of ligand binding protein, and these hydrogen bonds are beneficial to fix ligand in the process of Michael addition. Molecular dynamics help us to confirm our assumption about the catalytic reaction mechanism further and dynamically describe the catalytic process in comparison with the direct covalently docking method.

Since molecular dynamics can only simulate the trajectories of atoms but not bond break or formation, therefore this problem is needed to be explored by quantum chemistry. The issue we are concerned about is whether the sulfur atom of Cys85 and the 15-position olefin carbon of ligand can form a covalent bond at such stable distances from 3.45 to 3.80 Å. According to calculated results, the free energy barriers for the covalent attachment of Cys85 to the substrate are respectively 83.22, 79.55 and 73.86 kJ/mol. In comparison with UbcH5a and UbcH5b, UbcH5c with lower free energy barrier can effectively form the covalent bond. The energy barriers of the carboxyl hydrogen of Asp117 transferring to the 14-position carbonyl oxygen of ligand are 30.21, 29.20 and 27.50 kJ/mol respectively. Compared with UbcH5a and UbcH5b, the hydrogen of UbcH5c with lower free energy barrier transfers from Asp117 to 14-position carbonyl oxygen. According to the above analysis, the order of activation efficiency and effectiveness of covalent reaction can be sorted as UbcH5c > UbcH5b > UbcH5a. Cys85 of UbcH5c is more efficient to be activated and form a covalent bond with the ligand. The result is consistent with Zhenlin Hu and Weidong Zhang team’s research which has reported selectivity of among UbcH5a, UbcH5b, and UbcH5c with apparent dissociation constants (KD) of 5.189 mM, 3.578 mM, and 2.577 mM, respectively. Therefore, we infer that 1β-hydroxy alantolactone is more selective for UbcH5c.

In summary, we provide an interpretation for the mechanism of 1β-hydroxy alantolactone covalently bonds with UbcH5s. During this research, several residues including Asp112, Asn114 and Asp117, are particularly important, not only assisting in fixing ligand but also ensuring completion of the catalytic reaction. We confirmed that the molecular geometry of 1β-hydroxy alantolactone and the chemical environment around the target thiol group is the essential factors determining the reactive selectivity of ligand toward Cys85 of UbcH5s. 1β-hydroxy alantolactone represents the basis for a potential new class of therapeutics for anti-TNF-α interventions. A better understanding of the molecular mechanism for the ligand interacting UbcH5s can provide essential information about the development of ubiquitin-related drugs.

## Materials and Methods

### Sequence and structural analysis

In this research, amino acid sequences were aligned and 3D structures of UbcH5a (PDB ID: 3PTF), UbcH5b (PDB ID: 3EB6) and UbcH5c (PDB ID: 1 × 23) were superimposed. In addition, Ube2S (PDB ID: 1ZDN), UbcH7 (PDB ID: 1C4Z), Ubc1 (PDB ID: 1TTE), UbcH8 (PDB ID: 1WZV), Ube2B (PDB ID: 2YB6), Ube2J2 (PDB ID: 2F4W), Ubc7 (PDB ID: 2AW7), Ube2W (PDB ID: 2A7L) and CDC34 (PDB ID: 3RZ3) were downloaded to analyze residues distribution around Cys which is corresponded to Cys85 of UbcH5s. The organism of 3EB6 is Homo sapiens and Xenopus laevis, and the sequence is the same between humans and xenopus.

### Molecular docking

Autodock 4.2^37^ with Lamarckian genetic algorithm (LGA)^38^ was employed for the docking mode selection due to its good performance of reproduction capability^39,40^. Before molecular docking, the activated UbcH5s proteins in **(2)** step of the protocol were used to dock with 1β-hydroxy alantolactone. PDB2PQR Version 2.1.1^41^ was used to prepare proteins for the calculation of electrostatic potentials. Protonation states of ionizable side chains were obtained with the PROPKA 3.1 method^42,43^ implemented in the PDB2PQR server. AutoDockTools 1.5.6^44^ was used to prepare charge of proteins and save proteins as pdbqt file. The 3D structure of 1β-hydroxy alantolactone was constructed by ChemDraw ultra 2.0^45^, and VEGA ZZ 3.1.2^46^ was used to calculate and add MOPAC charge to ligand. Avogadro-1.2.0^47^ was utilized to optimize structure of 1β-hydroxy alantolactone with method of steepest descent and gradient descent. Then AutoDock Tools 1.5.6 prepared pdbqt file for subsequent docking program. The docking calculations were performed by locating a 40 × 40 × 40 points grid map and with a 0.375 Å grid point spacing which covered the catalytic sites. Each ligand was docked for 100 times to meet the demand of retrieving the top one docking poses.

### Molecular dynamics and reaction modeling studies

In this study, we firstly constructed two systems, which respectively were inactivated UbcH5s systems (**1**) and activated UbcH5s docked with ligands systems (**2**). Molecular dynamics was performed using the GROMACS 5.1.4. Amber99sb Force Field and the tip3p water condition were selected during the topology process. In this research, the above two kinds of systems were placed in the box at least 1.0 nm from the box edge, and the box was defined as a cube. We performed energy minimization to reach the maximum force below 250 kJ/mol. Equilibrating the water around the protein was performed under 100 ps NVT followed by 100 ps NPT ensembles at 298 K. MD data was collected for 100 ns in the NPT ensemble at 298 K, and the GROMACS program was used to constrain the bonds involving hydrogen atoms, allowing for an integration interval of 2 fs. A cutoff 1.0 nm was used for long-range van der Waals energies, and the particle mesh Ewald method^48^ was used for computing electrostatic interactions. The non-bonded pair lists were updated every 0.010 ps. The coordinates were saved every 100 ps.

To detect free energy barrier of Cys85 of UbcH5s covalently bonded with 1β-hydroxy alantolactone, we extracted respectively 10 snapshots of the dynamical trajectory from UbcH5a/b/c systems every 10 ns in the **(2)** step. Then quantum mechanics-molecular mechanics (QM/MM) potential was employed to calculate the energy barrier. QM/MM calculations were performed in Gaussian 09 using the ONIOM electronic embedding scheme^49^. QM region contained the ligand, Cys85 and Asp117, which were treated at the DFT B3LYP/6–31 G(d) level^50,51^. The surroundings were treated with MM region at UFF level. In addition, Mulliken charge has also been calculated to detect the electronic displacement. Then intermediates were used to MD simulation. The paraments of MD were consistent with the above dynamical description.

At the **(03)** step, the conformations of sulfur atom of Cys85 covalently bonded with ligands were used to MD simulation, and the paraments of MD were consistent with the above dynamical description. After 100 ns simulation, we extracted respectively 10 conformations from the dynamical trajectory of UbcH5a/b/c systems every 10 ns for the subsequent QM/MM calculation.

Gaussian 09 using the ONIOM electronic embedding scheme was performed to calculate QM/MM. Ligands, Cys85s and Asp117s included in QM region were treated at the DFT B3LYP/6–31 G(d) level. The surroundings of complexes were treated as MM region at UFF level. At **(4)** step, the calculated products sourced from 100 ns conformation were used for subsequent molecular dynamics. And paraments of MD was consistent with the above dynamical description.

### Alanine scanning mutagenesis

To evaluate the vital role played by the hotspot residues of UbcH5s interacting with 1β-hydroxy alantolactone, the snapshots of each complex every 5 ns were extracted to Alanine scanning mutagenesis by using the ABS-Scan web server for protein–ligand alanine scanning^52^. To visualize the contribution of each residue interacting with ligand, each mutated structure will be scored by using the empirical scoring function of AutoDock^53^.

### Data analysis

When the simulation was completed, the trajectories of each system were concatenated for analysis. PyMol (www.pymol.org) YASARA View^54^ and VMD 1.9.3^55^ were used to create images. All electrostatic surfaces were calculated using Multiwfn 3.7^56^ and images were rendered using PyMol and VMD 1.9.3. Multiple sequence alignments were performed using CLUSTALW and further analyses were performed using ESPript 3.0^57^. Root-mean-square deviation (RMSD) and root mean square fluctuation (RMSF) of backbone heavy atoms with respect to their initial structure were calculated every 100 ps. In the catalytic site, several distances between Cys85 and Asp117, ligand and Asp112, ligand and Asp117 and ligand and Asn114 were analyzed by using Gromacs. Binding energies were calculated by using the MM-PBSA tool as described by Kumari *et al*.^58^, utilizing APBS^59^ to solve Possion-Boltzman equations. The extensive details have been described in the paper and on the web (http://rashmikumari.github.io/g_mmpbsa/accessed.21040920). In addition, hydrogen bond changes between ligand and protein, hydrophobic strength between ligand and protein were also analyzed by Gromacs.

## Supplementary information

Supplementary Information.
