# Supplementary material for: Molecular Simulation Elaborating the Mechanism of 1β-Hydroxy Alantolactone Inhibiting Ubiquitin-Conjugating Enzyme UbcH5s
Source: Sci Rep. 2020 Jan 10;10:141. doi: 10.1038/s41598-019-57104-4 (PMC6954291; doi:10.1038/s41598-019-57104-4)
Supplement: Supplementary file 1 — Supplementary Information. [file 41598_2019_57104_MOESM1_ESM.docx]

Supplemental Information

Molecular Simulation Elaborating the Mechanism of 1β-Hydroxy Alantolactone Inhibiting Ubiquitin-Conjugating Enzyme UbcH5s

Youdong Xu, Xianli Meng

Supplemental Data


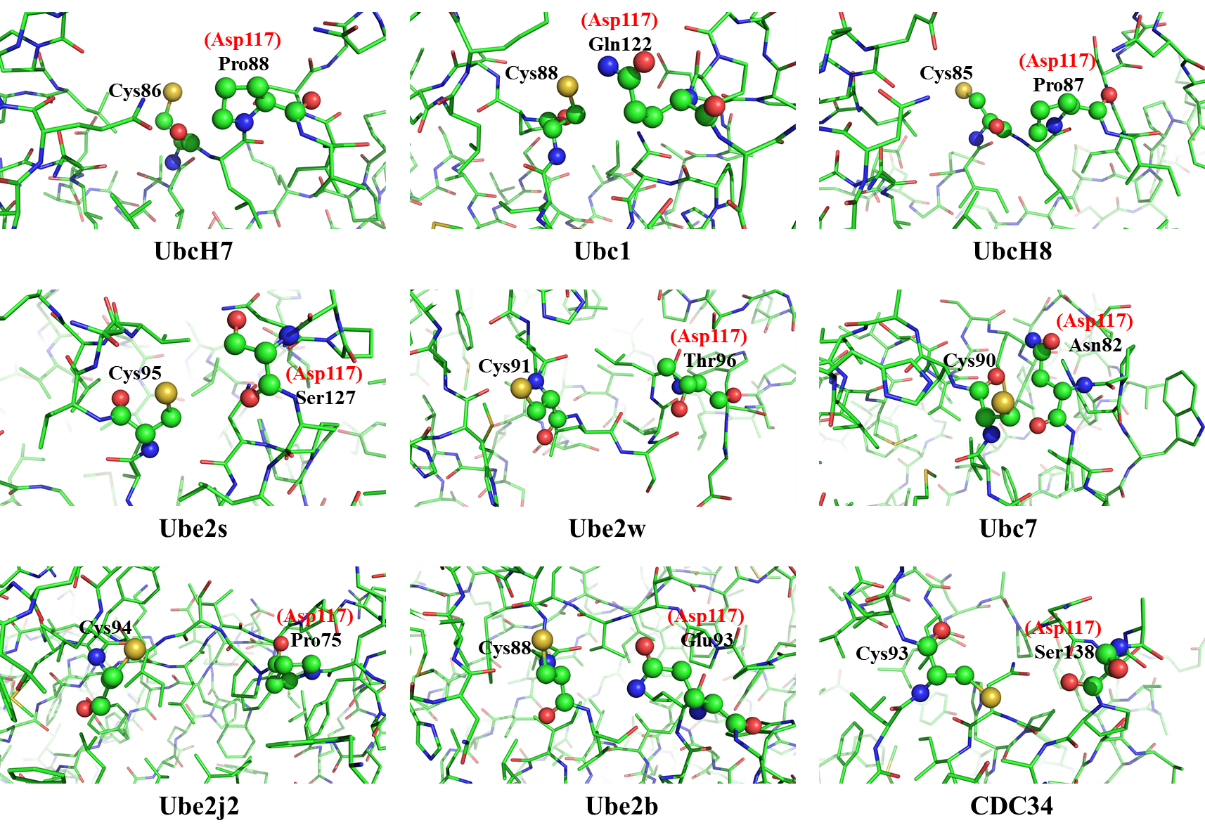


Figure S1. In these proteins, only Cys was found, but no Asp which could form catalytic diad with Cys was found. To further explain the issue, the amino acids at 3D positions equivalent to Asp117 are also highlighted. Cys and these amino acids are showed as ball stick model, rest residues are showed as stick model. Red font “(Asp117)” was used to emphasize that these amino acids are equivalent to the 3D position of Asp117.


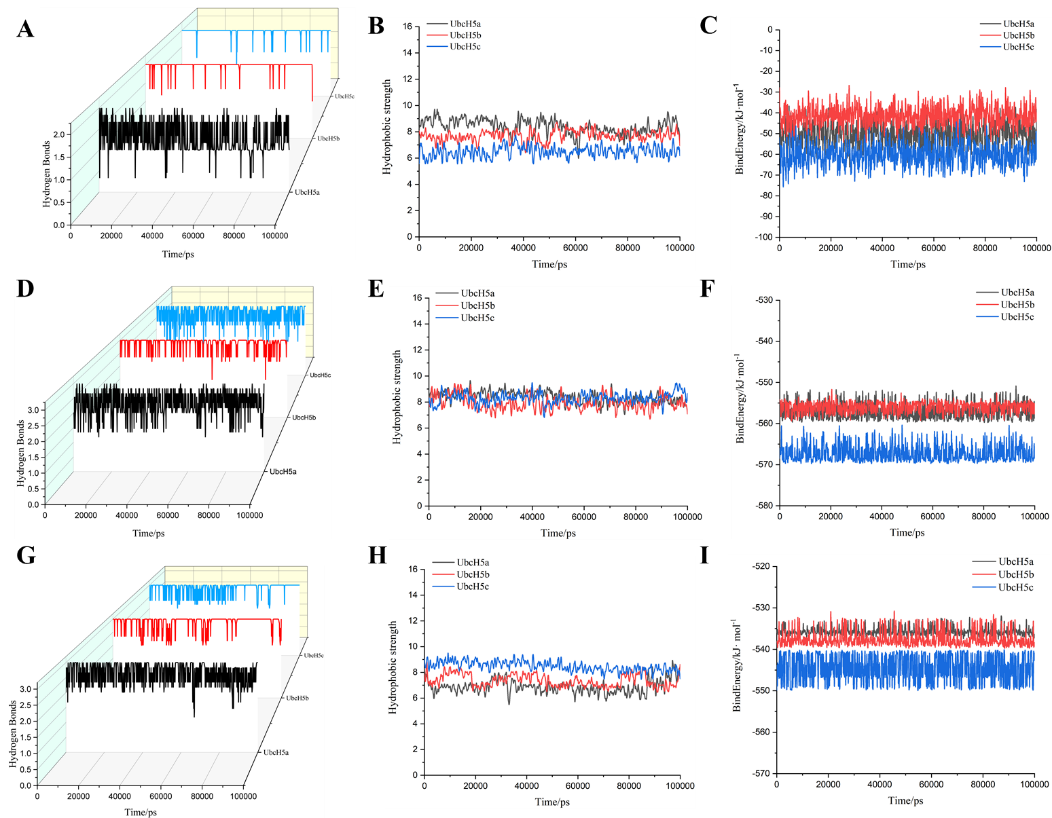


Figure S2. Waterfall maps which are used to describe number of hydrogen bonds between residues of UbcH5s and ligand. (A) The number of hydrogen bonds between 16-position hydroxyl hydrogen of ligand and oxygen of Asp112, 13-position epoxy and amino hydrogen of Asn114; (D) The number of hydrogen bonds between 14-position carbonyl oxygen of ligand and carboxyl hydrogen of Asp117, 16-position hydroxyl hydrogen of ligand and oxygen of Asp112 and 13-position epoxy and amino hydrogen of Asn114; (G) The number of hydrogen bonds between 14-position hydroxyl hydrogen of ligand and carboxyloxy of Asp117, 16-position hydroxyl hydrogen of ligand and oxygen of Asp112 and 13-position epoxy and amino hydrogen of Asn114; (B), (E) and (H) are hydrophobic strength between ligand and proteins; (C), (F) and (I) are binding energy between ligand and protein.


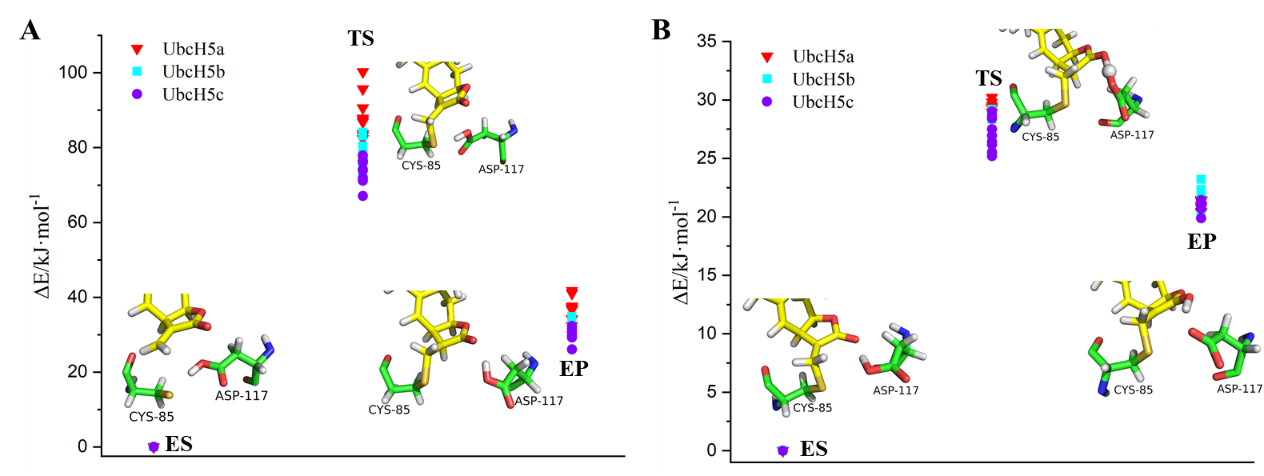


Figure S3. The calculated free energy barriers for 1β-hydroxy alantolactone forming covalent bond with Cys85 of UbcH5s and hydrogen transfer by using QM (DFT B3LYP/ 6-31G(d))/MM (UFF). (A) The calculated free energy barriers for ligands covalently bonding with Cys85, and the 30 conformations extracted dynamical trajectory of **(2)** systems. (B) The calculated free energy barriers for hydrogen transferred from Asp117’s carboxyl to ligand’s 14-position carbonyl oxygen, and the 30 conformations extracted dynamical trajectory of **(3)** systems. Red inverted triangles, cyan square and purple circle represent respectively the energy barrier of UbcH5a/b/c. Cys85 and Asp117 are displayed as green stick model and 1β-hydroxy alantolactone showed as yellow stick model. For easy identification, the ligand is partially displayed.

Figure S4. The distribution of charge at 11-position carbon and 14-position oxygen of ligand after formation of covalent bond.


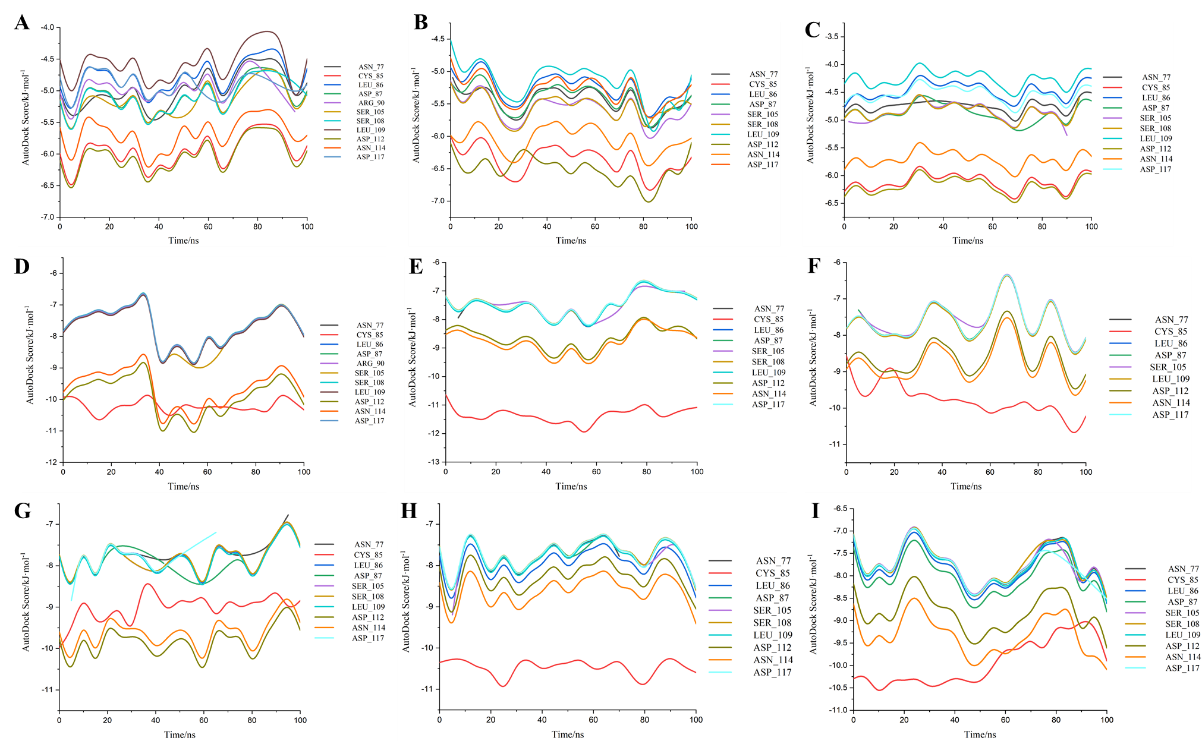


Figure S5. The energetic contribution of residues for docking with ligand, (A)-(C) When Ligand docked with protein, the energetic contribution of residues of UbcH5a/b/c for ligand; (D)-(F) When carbon atom at 15 position of ligand covalently bonding with sulfur, the energetic contribution of residues of UbcH5a/b/c for ligand; (G)-(I) When carboxyl hydrogen of Asp117 transferred to carbonyl oxygen at 14 position of ligand, the energetic contribution of residues of UbcH5a/b/c for ligand. Because of energy contribution changes over time, the lower the energy curve, the greater the energy contribution.
